# Morphological Diversity and Evolution of Jaw Morphologies in Zeiform Fishes (Teleostei, Paracanthopterygii)

**DOI:** 10.1093/iob/obae011

**Published:** 2024-04-15

**Authors:** J W Peters, K K Duclos, M V H Wilson, T C Grande

**Affiliations:** D epartment of Biology, Loyola University Chicago, Chicago, IL 60660, USA; Department of Cell Biology and Anatomy, The University of Calgary, Calgary T2N 1N4 Alberta, Canada; D epartment of Biology, Loyola University Chicago, Chicago, IL 60660, USA; D epartment of Biology, Loyola University Chicago, Chicago, IL 60660, USA

## Abstract

Zeiformes (dories, tinselfishes, and oreos) are primarily benthopelagic acanthomorph fishes, distributed between 50 and 1000 m depth on continental slopes and on flanks of oceanic islands and seamounts. Among the interesting morphological adaptations of zeiform fishes are their unique and highly protrusible jaws involving premaxillae with long ascending processes and a four-bar linkage, including mobile palatines that pivot on their posterior articulation. This adaptation for increased jaw protrusion has enabled zeiform fishes to capture elusive prey more efficiently and is arguably a major factor in their morphological diversity and evolutionary success. This study examines the evolution of zeiform jaw morphologies using 3D landmark-based multivariate morphometrics as well as phylomorphospace analysis. Results show that the descendants of the zeiform ancestor branched rapidly early in their history, retaining conservative jaw morphologies during this early branching, but subsequently strongly diverged in many of the resulting lineages. Results from this study are compared with earlier research based on overall body form, demonstrating that morphological variation within Zeiformes arose along at least two distinct trajectories: body form and jaw morphology. Variation among genera in body form is not associated with variation among the same genera in jaw morphology, and vice versa. Hypotheses to explain the apparent decoupling of body shape and jaw morphology are addressed along with avenues for further study to better understand the morphological evolution of these iconic fishes.

## French Language Translation

Les Zéiformes (e.g., dorés, oréos et poissons scintillants) sont des poissons benthopélagiques du groupe des Acanthomorpha trouvés entre 50 et 1000m de profondeurs sur les talus continentaux, et sur les abords des iles océaniques et des monts sous-marins. Parmi les adaptations morphologiques des zéiformes figurent leur mâchoires protrusibles, composées de prémaxillaires avec de longs processus ascendants et un mécanisme à quatre barres qui implique des os palatins mobiles qui pivotent autour de leur articulation postérieure. Ces adaptations morphologiques conduisent à une protrusion buccale sur de plus grandes distances et plus efficace, permettant aux zéiformes de capturer des proies autrement insaisissables et sont des éléments majeurs de la diversité morphologique du groupe ainsi que de leur succès évolutif. Cette étude examine l’évolution de la morphologie des mâchoires des zéiformes via des mesures de morphométrie géométrique 3D par points d'intérêts et des analyses d'espace phylo-morphologique. Les résultats montrent que les Zéiformes se sont rapidement séparés du plus vieil ancêtre commun mais ont initialement conservés des morphologies de mâchoires similaires. Suite à cette séparation initiale, les morphologies ont rapidement divergé menant à la diversité courante du groupe. Une comparaison des résultats obtenus ici avec des études précédentes sur la forme du corps des Zéiformes soutient que l’évolution morphologique du groupe a suivi au moins deux axes distincts : la forme du corps et la morphologie des mâchoires. Les différences de formes du corps entre genres ne sont pas corrélées avec les différences de formes de mâchoires entre ces mêmes genres, ni réciproquement. Des hypothèses quant au découplage entre la forme du corps et la morphologie des mâchoires sont avancées et plusieurs pistes de recherche future pour nous permettre de mieux comprendre l’évolution morphologique de ce groupe de poissons fascinant sont proposées.

## Spanish Language Translation

Los zeiformes (dorios, peces oropel y oreos) son peces acantomorfos principalmente bentopelágicos, los cuales se encuentran distribuidos entre 50 y 1000 m de profundidad en los taludes continentales, así como en los flancos de islas oceánicas y montes submarinos. Estos peces poseen adaptaciones morfológicas particulares, entre las cuales se incluye la presencia de una mandíbula altamente protusible incluyendo premaxilares con procesos ascendentes largos, y una articulación de 4-barras con palatinos móviles que pivotean sobre su articulación posterior. Esta adaptación en la protrusión mandibular ha permitido a los zeiformes capturar presas elusivas de manera más eficiente, y posiblemente ha sido un factor importante en su diversidad morfológica y éxito evolutivo. El presente estudio examina la evolución de la morfología de la mandíbula de los peces zeiformes utilizando un análisis morfométrico multivariado basado en puntos de referencia (3D landmark-based), así como un análisis de tipo filomorfoespacial. Los resultados de este trabajo muestran que los zeiformes se diversificaron rápidamente en su historia temprana, manteniendo la morfología de la mandíbula inicial durante esta etapa. No obstante, posteriormente este grupo de peces se diversificaron considerablemente en diferentes linajes. Asimismo, los resultados obtenidos se compararon con investigaciones anteriores basadas en la forma general del cuerpo, lo que demuestra que la variación morfológica de los zeiformes surgió a lo largo de al menos dos rasgos morfológicos distintos: la forma del cuerpo y la morfología de la mandíbula. Con relación a esto, se encontró que la variación de la forma del cuerpo no está asociada con la variación de la morfología de la mandíbula entre los mismos géneros de los peces zeifomes, y viceversa. Finalmente, este estudio aborda las hipótesis que explican la aparente disociación entre la forma del cuerpo y la morfología de la mandíbula de este grupo de peces, así como las rutas para estudios posteriores que ayude a comprender mejor la evolución morfológica de estos icónicos peces.

## Introduction

**Fig. 1:**
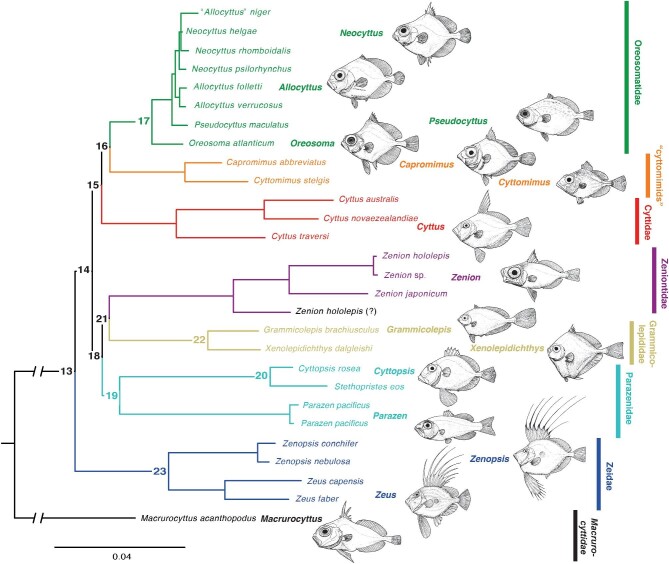
Phylogeny of Zeiformes from Grande et al. (2018: fig. 7), based on Bayesian Inference of combined morphological and molecular evidence. Names of extant family-level clades are shown at right. The “cyttomimids,” formerly in Zeniontidae, are an unnamed family-level clade indicated by the results of Grande et al. (2018) but requiring further study. Drawings of representative fishes are by Michael Hanson. Numbered ancestral nodes are referred to in the section on the phylomorphospace, below.

Zeiformes (e.g., dories, lookdown dories, tinselfishes, and oreos) are a primarily benthopelagic group of acanthomorph fishes living and foraging near the sea floor at depths of 50–1000 m. They have a global tropical and temperate distribution with some species having near-worldwide ranges, while others are regional endemics (such as in the waters off New Zealand and Australia). Zeiformes are classified within Paracanthopterygii, sister to Stylephoriformes + Gadiformes (Betancur-R et al. 2013; Grande et al. 2013), and have a fossil record dating back to the late Campanian/early Maastrichtian, or ∼72 Mya (Tyler and Santini 2005; Davesne et al. 2017; Hughes et al. 2018; Ghezelayagh et al. 2021). Molecular studies estimate that the last zeiform–gadiform common ancestor could be as old as early/middle Campanian (81 Mya; Hughes et al. 2018), while crown-group zeiforms are estimated to have radiated rapidly as recently as the early Eocene (50–55 Mya; Ghezelayagh et al. 2021). The rapid radiation of the crown group is reflected in short branch lengths uniting most of the extant families (Fig. 1: nodes 14–19, 21).

The order (Fig. 1) currently consists of 33 extant species in 16 genera (Nelson et al. 2016), recognized in seven family-level clades based on work most recently by Grande et al. (2018): Macrurocyttidae (*Macrurocyttus*, formerly as Macrurocyttinae in Grammicolepididae), Zeidae (*Zeus* + *Zenopsis*), Parazenidae (*Parazen* + [*Cyttopsis* + *Stethopristes*]), Grammicolepididae (*Grammicolepis* + *Xenolepidichthys*), Zeniontidae (*Zenion*), Cyttidae (*Cyttus*), “cyttomimids” (*Cyttomimus* + *Capromimus*, an unnamed clade formerly in Zeniontidae), and Oreosomatidae (*Allocyttus, Neocyttus, Oreosoma*, and *Pseudocyttus*).

Zeiforms are of great interest not only because of their position as early evolving acanthomorphs, but also because of their unique and highly protrusible jaws employing premaxillae with greatly elongated ascending processes and large articular and postmaxillary processes (Heemstra 1980; Tyler et al. 2003; Westneat 2004; Davesne et al. 2017; Peters 2023). They also have mobile palatines that pivot on their posterior joint with the rest of the suspensorium (Tyler et al. 2003; Westneat 2004). This adaptation for increased upper jaw protrusion allows zeiforms to capture more elusive prey, such as small crustaceans and fishes, from a near-stationary position, and is considered to be an important factor in their evolutionary success (Staab et al. 2012; Bellwood et al. 2015).

Westneat (2004) surveyed acanthomorph jaw mechanisms and included *Cyttopsis rosea* as the model for Zeiformes in his study. He drew attention to its upper jaw protrusion, controlled by a unique four-bar linkage mechanism (illustrated here in Supplementary Fig. S1 and Supplementary Videos S1–S3). Westneat (2004) argued that this jaw mechanism arose within Zeiformes, and that analogous mechanisms evolved convergently among other spiny-rayed fishes. Each of the four bars in the zeiform mechanism has a role. The mandible—the input link—articulates posteriorly at the quadrate-articular joint with the fixed link (the suspensorium + braincase) and, by short ligaments at its coronoid process, to the distal end of the maxilla. The maxilla—the coupler link—rotates about its anterodorsal joint with the palatine—the output link. However, unlike the fixed palatines of most other fishes, the palatine pivots about its posterior joint with the lateral ethmoid + suspensorium (Tyler et al. 2003). Thus, three of the links—mandible, palatine, and maxilla—are moveable (in some non-zeiform taxa, the nasal, instead of the palatine, can be moveable). Change in the angles at any of the four joints causes compensating changes at the remaining joints (Westneat 1990). The premaxilla is not one of the four links; however, pivoting of the palatine and rotation of the maxilla cause the premaxilla to slide forward along its ascending process, increasing the degree of upper jaw protrusion (Westneat 2004).

Despite sharing the four-bar linkage mechanism of protrusion, zeiforms exhibit considerable variation in their jaw morphologies as evidenced by the acquisition of numerous disparate morphologies over a short period of time (Grande et al. 2018). For example, Cyttidae (lookdown or big-eye dories) are large-bodied fishes with large mouths and long ascending premaxillary processes, while Parazenidae (slender or smooth dories) exhibit a more elongate body form with large and oblique jaws and shorter ascending processes. Thus, the overall goal of this study is to describe the jaw morphologies and the key differences among the zeiform taxa.

This study differs from previous studies (e.g., Tyler et al. 2003; Westneat 2004) in its taxon breadth, with the inclusion of previously unavailable specimens, in its use of X-ray micro-computed tomography (µ-CT) allowing study of rare or unique specimens, and its employment of three-dimensional geometric morphometrics. Specific objectives of this study are to describe the morphological diversity of zeiform jaws and to examine phylogenetic changes or trajectories in the evolution of jaw morphologies by reconstructing the ancestral states at various points in the phylogeny under phylomorphospace analysis. We conclude with a discussion of evolutionary trajectories, interpretations of and hypotheses (modularity, strong selection, etc.) for the observed variation, along with suggested avenues for further study.

## Materials and methods

### Materials examined

All zeiform family-level clades are represented here except Macrurocyttidae, which is known only from larval or early post-larval specimens (Grande et al. 2018). Specimens of 11 of the 16 recognized genera were available for this study. The rarity of suitable specimens in museum collections prohibited the inclusion of *Neocyttus, Pseudocyttus*, and *Cyttomimus*, for which only DNA samples were available (Grande et al. 2018). In the case of *Macrurocyttus*, neither DNA nor intact adult specimens were available (Tyler et al. 2003; Grande et al. 2018).

All included specimens had fully ossified axial skeletons, ranging from 42 to 168 mm SL, almost all with fully closed jaws, and all were preserved in 75% ethanol. The anterior portions of adult specimens were scanned using a Perkin Elmer Quantum GX2 µ-CT scanner (High-resolution scans, Copper/Aluminum filter) and exported as DICOM stacks for further analysis. An additional scan of *Grammicolepis brachiusculus* was obtained from the University of Florida Museum of Natural History Division of Ichthyology through the data repository Morphosource (UF: Fish:124132, http://n2t.net/ark:/87602/m4/437933), under the BY-NC-SA licence.

The materials examined are listed below and use the following institutional abbreviations: FMNH, Field Museum of Natural History; KU, University of Kansas Museum of Natural History; LACM, Natural History Museum of Los Angeles County; LUC, Loyola University Chicago, Department of Biology; MCZ, Museum of Comparative Zoology, Harvard University; and USNM, Smithsonian National Museum of Natural History.

**Parazenidae*.—*** *Parazen pacificus*: 5 specimens, 95.2–100.2 mm SL: FMNH 64402, 64403, 65401, 67158; USNM 364277.

*Cyttopsis rosea*: 6 specimens, 62.2–143.8 mm SL: FMNH 67901, 67093, 67095, 67097; USNM 377980.

*Stethopristes eos*: 1 specimen, 105.2 mm SL: USNM 226570.

**Zeidae*.—*** *Zenopsis conchifer*: 13 specimens, 42.3–105.4 mm SL: FMNH 67090, 67179, USNM 159819, 372241.

*Zeus faber*: 4 specimens, 76–113 mm SL: FMNH 4062; USNM 307842, 325986.

**Zeniontidae*.—*** *Zenion hololepis*: 4 specimens, 60.3–84.4 mm SL: FMNH 64410; USNM 377986.

“**cyttomimids**”*—Capromimus abbreviatus*: 5 specimens, 71–83 mm SL: LUC 1308.1–5.

**Grammicolepididae*.—*** *Xenolepidichthys dalgleishi*: 4 specimens, 55–78.2 mm SL: FMNH 74320.

*Grammicolepis brachiusculus*: 1 specimen, UF: Fish: 124132 (scan only; see above).

**Cyttidae.** *—Cyttus australis*: 1 specimen, 167.9 mm SL: MCZ 17264.

**Oreosomatidae.** *—Oreosoma atlanticum*: 1 specimen, 127.6 mm SL: KU 33415.

*Allocyttus verrucosus*: 1 specimen, 142.2 mm SL: LACM 44752.

### Morphological analyses

**Fig. 2:**
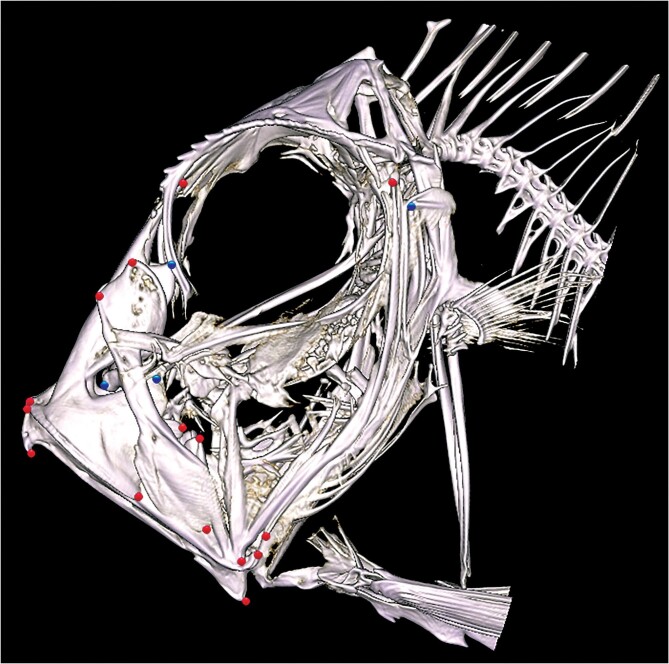
2-D image in lateral view of an isosurface µ-CT reconstruction (Fedorov et al. 2012) of *C. rosea* (FMNH 67,093), showing the external (red) and internal (blue) landmarks.

**Fig. 3:**
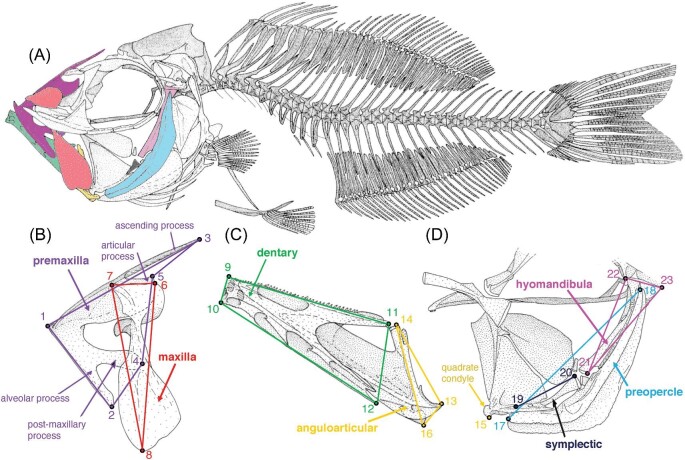
Skeleton of *P. pacificus* in lateral view, from Tyler et al. (2003), California Academy of Sciences CAS 38,404, 96 mm SL, showing landmarked bones and landmark locations. (A) Drawing of the entire skeleton of *P. pacificus* showing the positions of the landmarked bones. (B) Landmarks 1–8 of the upper jaw (premaxilla and maxilla). (C) Landmarks 9–16 of the lower jaw (dentary and anguloarticular). (D) Landmarks 17–23 of the suspensorium (symplectic, hyomandibula, and preopercle). All drawings are modified after Tyler et al. (2003). For definitions of specific landmarks, see Table 1. The individual bones are as follows: anguloarticular, yellow; dentary, green; hyomandibula, violet; maxilla, red; premaxilla, purple; preopercle, pale blue; and symplectic, black.

Scans were imported into Stratovan Checkpoint 2018 (Version 2018.08.07) and visualized as isosurfaces using a density threshold (Fig. 2). Twenty-three homologous landmarks (Table 1, Fig. 3, Supplementary Fig. S2) were then digitized in 3-D across all specimens. Landmarks (Table 1) were chosen to best capture mechanically relevant shape information of the oral jaws plus posterior suspensorium, and to describe the premaxilla, maxilla, dentary, and anguloarticular of the jaws and the symplectic, preopercle, and hyomandibula of the suspensorium, with the constraint that landmarks be recognizable and homologous on all specimens. Detailed descriptions of zeiform jaw anatomy from Tyler et al. (2003) and direct examination of cleared and stained specimens guided landmark decisions.

**Table 1:** List of the 23 landmarks and their definitions

Landmark number	Landmark description
1	Anterior tip of premaxilla
2	Posteroventral tip of alveolar process of premaxilla
3	Dorsal tip of ascending process of premaxilla
4	Posterior extent of postmaxillary process of premaxilla
5	Posterodorsal tip of articular process of premaxilla
6	Posterodorsal tip of maxilla
7	Anterodorsal tip of maxilla
8	Posteroventral tip of maxilla
9	Anterodorsal tip of oral margin of dentary
10	Anteroventral tip of symphysis of dentary
11	Posterodorsal tip of dorsal ramus of dentary
12	Posteroventral tip of ventral ramus of dentary
13	Posterior tip of anguloarticular
14	Dorsal tip of anguloarticular
15	Base of quadrate condyle
16	Posterior extent of retroarticular process of anguloarticular
17	Ventral tip of preopercle
18	Dorsal tip of preopercle
19	Anteroventral tip of symplectic
20	Dorsal tip of symplectic
21	Ventral tip of shaft of hyomandibula
22	Anterodorsal tip of condyle of hyomandibula
23	Posterodorsal tip of condyle of hyomandibula

*Note*: Landmarks were placed unilaterally.

### Statistical analysis

Landmark coordinates were subjected to a generalized Procrustes superimposition using the gpagen function in geomorph (Baken et al. 2021; Adams et al. 2022). Museum specimens are not standardized in their preservation and some specimens can be preserved with open mouths, which might affect morphological analysis (Gartner et al. 2023). To address this, we estimated “gape” as the ratio of the distance between landmarks one and nine to skull length (as the distance between landmarks 1 and 18). We found two specimens for which that ratio was above 10%, *Cyttus australis* (MCZ 17264: 10.4%) and *Stethopristes eos* (USNM 226570: 14.6%). Jaw position should not significantly impact the description of the morphology of these two taxa; nevertheless, jaw positioning might theoretically affect principal components and might change the reconstruction of ancestral morphologies. However, Gartner et al. (2023) had more specimens with larger gapes (up to 24%), yet excluding those specimens had negligible effect on their results. Because the two specimens in our analyses are the sole representatives for their respective species and genera, and the amount of gape was relatively small, we did not remove them from our dataset.

We used principal components analysis (PCA) to visually assess and describe morphological differences and similarities across Zeiformes. All species were classified by family-level clades following the most recent phylogeny (Grande et al. 2018: fig. 7; Fig. 1). Shape changes corresponding to the first three principal components were visualized to describe the major aspects of shape variation within Zeiformes. We then calculated average shapes for individual species and generated a phylomorphospace (e.g., Sidlauskas 2008; Klingenberg and Marugán-Lobón 2013) based on the Bayesian-inference combined-evidence phylogeny from Grande et al. (2018) and the landmark data for jaws and posterior suspensorium (referred to hereafter as the jaw phylomorphospace). Because the tree from Grande et al. (2018) was not time calibrated, we forced the tree to be ultrametric using the *chronos* function from the R package ape (Paradis and Schliep 2019). The *gm.prcomp* function in geomorph allows for the reconstruction of ancestral morphologies of multiple nodes in time-calibrated phylogenies. We reconstructed the morphology of 11 ancestral nodes (including the root of the tree) to visualize how shape has changed throughout the branches of the zeiform phylogeny. All data analyses were performed in the R statistical programming environment, using the packages “Geomorph” (v4.0), ape, and abind (Plate and Heiberger 2016; Paradis and Schliep 2019; Baken et al. 2021; Adams et al. 2022).

## Results

### Variation in jaw morphology

**Fig. 4:**
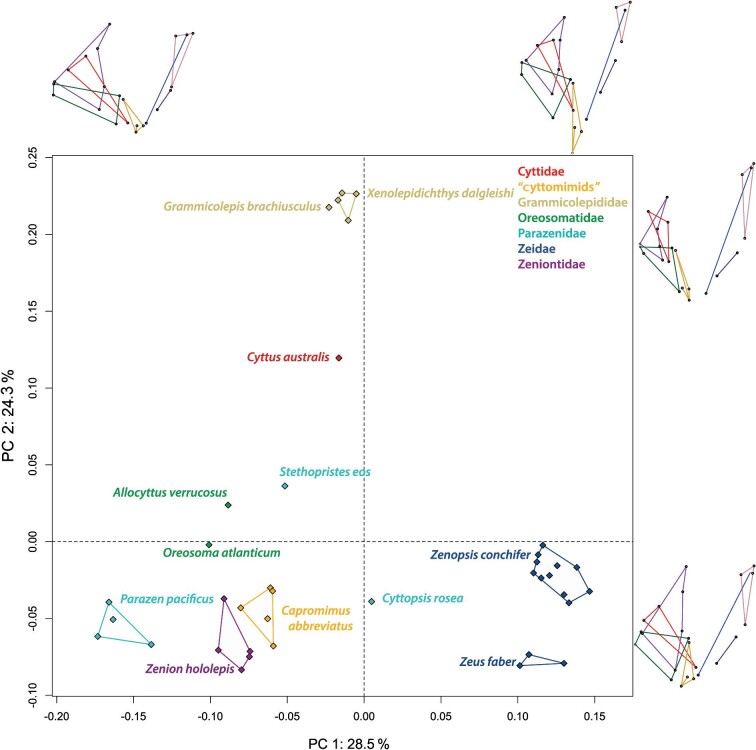
Results of the PCA of the 23 landmarks for 37 zeiform specimens (12 genera representing seven family-level clades). The *x*-axis represents PC1 and the *y*-axis represents PC2. Each of seven family-level clades as recognized by Grande et al. (2018) is given in the inset key. Wireframes show the shapes for the minimum and maximum values along each principal component.

**Fig. 5:**
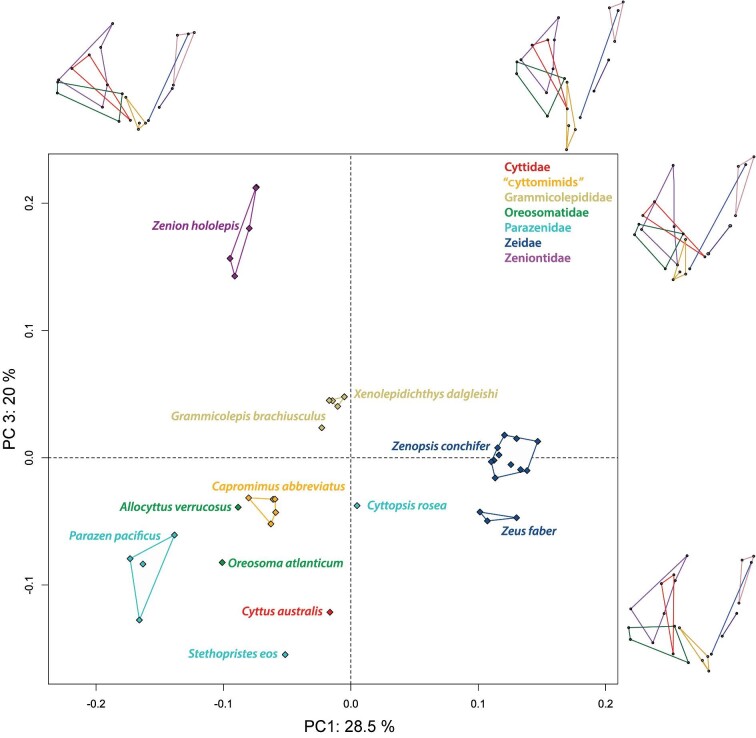
Results of the PCA for 23 landmarks on 37 Zeiform specimens. The *x*-axis shows PC1, while the *y*-axis shows PC3. Each represented family-level clade as recognized by Grande et al. (2018) is specified by color as given in the inset key. Wireframes show the shapes for the minimum and maximum values along each principal component.

Results of the PCA of Procrustes coordinates from 23 landmarks (Table 1) on 37 specimens, representing the studied zeiform family-level clades, are shown in Figs. 4 and 5. The first three principal components together explain more than 73% of the total variance (principal component 1 [PC1] 28.5%, principal component 2 [PC2] 24.3%, and principal component 3 [PC3] 20%). The plot of PC1 vs. PC2 shows strong species grouping without any outliers (Fig. 4). *Cyttopsis rosea* falls near the center of both principal components one and two, displaying a near average jaw shape for these components.

*Parazen pacificus* is representative of the maximum variation in the negative direction of PC1, whereas *Zenopsis conchifer* represents the maximum variation in the positive direction of PC1. For PC2, *Xenolepidichthys dalgleishi* illustrates the maximum variation in the positive direction, while *Zenion hololepis* and *Zeus faber* illustrate the maximum variation in the negative direction.

The wireframes (Fig. 4) illustrate that the first component corresponds to differences in overall jaw length and height, with a shift from the minimum PC1 capturing longer and vertically shorter jaws to maximum PC1 representing a taller and antero-posteriorly shorter jaw shape. The second principal component corresponds to a change in the relative length and proportions of the jaws, with the maximum negative variation of PC2 corresponding to larger jaws. The wireframes for PC1 and PC2 also capture a change in the relative size and length of the premaxillary bones (Fig. 4), with an increased premaxillary ascending process at the minimum of PC1 and a decrease in size and length of the ascending process at the minimum of PC2.

For PC3 (Fig. 5), *Stethopristes eos, Cyttus australis*, and *Parazen pacificus* are representative of the maximum variation in the negative direction, while *Zenion hololepis* illustrates the maximum variation in the positive direction (Fig. 5). The wireframes for PC3 reveal smaller changes in the orientation of specific bones of the jaws, but significant variation in the bones of the suspensorium (Fig. 5). PC3 also appears to capture jaw gape to a small extent (Fig. 5). Both *S. eos* and *C. australis*, for which >10% gape was estimated, occupy some of the most negative scores for PC3, but intermediate scores for PC1 and PC2. However, even those negative PC3 scores are similar to scores for *P. pacificus*, which did not have large gape scores.

For the maxilla, which is an important link in the four-bar feeding mechanism, PC1 shows a reduction in size and a change in angle at the maximum values (Fig. 4). The minimum PC2 variation displays a longer shape, and the maximum shows a change in angle and decrease in size. As was observed in PC2 for the premaxilla, there is a large decrease in the size and length of the maxilla corresponding to *Xenolepidichthys dalgleishi* and *Grammicolepis brachiusculus*. PC3 also captures a large change in the angle of the maxilla.

The dentary and anguloarticular bones show variation in the lower jaw, which is the input linkage for the four-bar feeding mechanism. Wireframe diagrams for PC1 of the dentary illustrate a difference in length and depth at its posterior end, together with a change in the length of the anguloarticular. The PC1 minimum for *Parazen pacificus* shows longer and more slender lower jaw bones vs. the PC1 maximum for *Zenopsis conchifer*, which has antero-posteriorly shorter and taller jaws. PC2 shows a drastic shift in the angle and length of the dorsal ramus of the dentary (landmark #11). PC3 also reveals changes to the shape and angle of the dentary.

In the suspensorium, which suspends the jaws from the neurocranium and is represented here by the hyomandibula, symplectic, and preopercle, there are notable changes in PC1. In PC2 and PC3, a small change in orientation and shape is seen. Of the individual bones of the suspensorium, the hyomandibula showed the most significant changes to its size and relative position in PC1, being vertically much shorter and more dorsally positioned at positive values, while the preopercle changes in its orientation and length.

Overall, PC1 captured the greatest amount of jaw variation, showing a shift from longer and more slender jaws in species such as *P. pacificus* to shorter, taller jaws in species such as *Z. conchifer*. PC2 captured a significant change in the size of the anterior portion of the jaws and skull. It also revealed large shifts in the size of the upper jaw bones, especially in the size of the premaxilla. In the lower jaw bones, it was possible to see variation in the proportions of the dentary and anguloarticular. PC3 also captured significant variation in the size and shape of the bones of the suspensorium.

### Phylomorphospace results

**Fig. 6:**
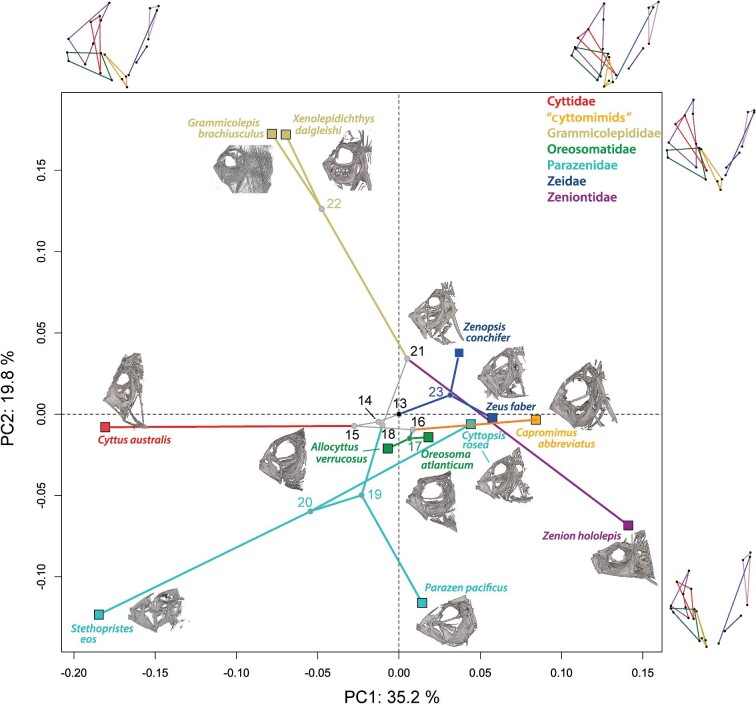
Combined-evidence jaw phylomorphospace. Morphometric analysis of average shapes for 12 study species plus 11 reconstructed ancestors (rather than the individual specimen data used for the earlier PCA in Figs. 4 and 5) plotted against an ultrametric phylogenetic tree. The tree is derived from the Bayesian combined-evidence phylogeny of Grande et al. (2018: fig. 7) presented in Fig. 1, which we forced to be ultrametric. The seven family-level clades are coded by color as shown in the insert. The black dot at the center (0,0) is the root of the tree of studied specimens, node 13 in Fig. 1. Wireframes show the shapes for the minimum and maximum values along each principal component. More detailed 2-D images of the scanned skulls are given in Supplementary Fig. S3.

Results from the phylomorphospace analysis (Fig. 6) show that the first two principal components (35.2 and 19.8%) account for 55% of total variance. This PCA differs from that of Figs. 4 and 5 in that this one is calculated not only from the landmarks of the extant taxa, but also from the reconstructed landmarks of their ancestors.

**Fig. 7:**
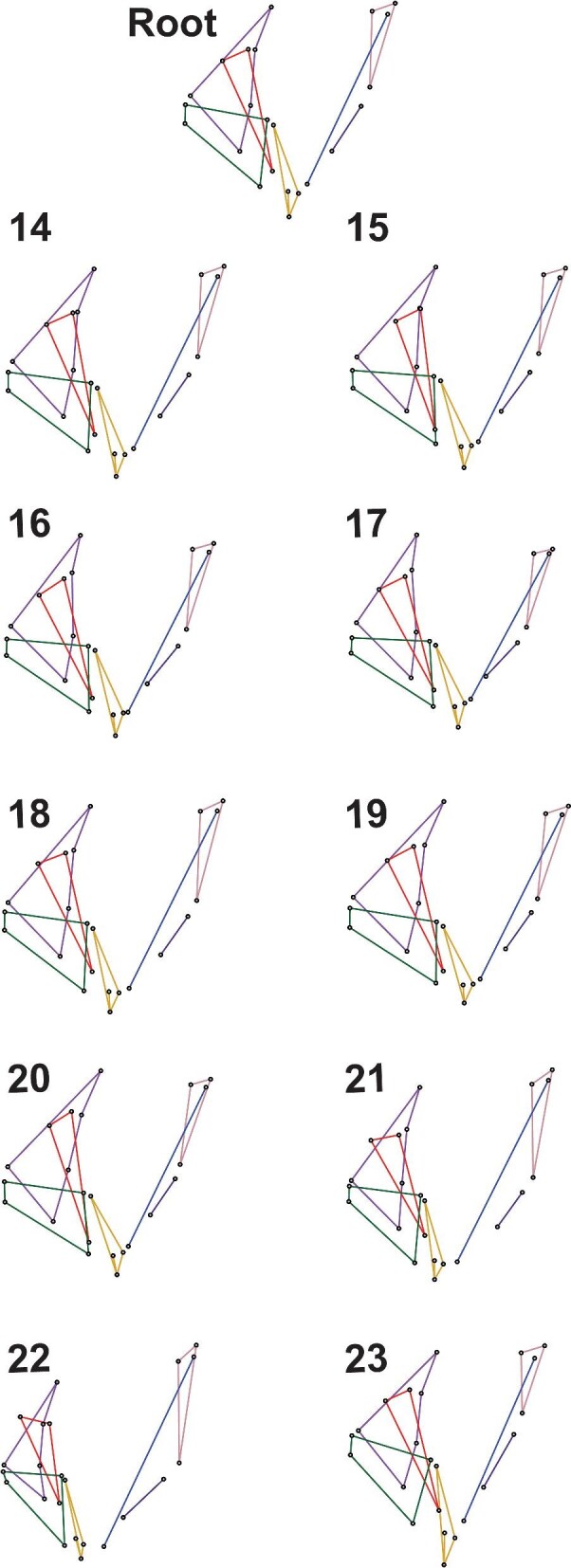
Wireframes for the reconstructed root and ancestral nodes 14–23 of the phylomorphospace. Node numbering follows Figs. 1 and 6. See text for details.

The results indicate that early branching from the root (node 13) led to ancestral jaw morphologies that were similar across ancestors for all zeiform families. Indeed, nodes 14–18 cluster relatively tightly together near the center of the phylomorphospace (Fig. 6). One family separates early from the last common ancestor of all 12 studied zeiform genera (node 13; Fig. 7: Root): the Zeidae, represented by node 23 and descendants *Zenopsis conchifer* and *Zeus faber*. However, this lineage does not diverge strongly in the first two principal components. Its sister-lineage leads to the last common ancestor of all other zeiforms (Fig. 7: node 14). The morphological differences between nodes 13 and 14 are very few, but the last common ancestor of the two genera of Zeidae (node 23) presents differences from the root at node 13. For example, in Zeidae, the ascending process of the premaxilla is inclined more posteriorly. The angles of both the alveolar process of the premaxilla and of the dentary also change, both becoming more steeply inclined when the jaws are closed. In the lower jaw, the dentary becomes dorso-ventrally deeper posteriorly, and the anguloarticular extends more ventrally. In the suspensorium, as illustrated by Tyler et al. (2003, Fig. 73), the preopercle appears relatively longer at node 23 than at the root node (Fig. 7). In addition, the hyomandibula is positioned relatively more dorsally and has a relatively much shorter main shaft, which articulates with an elongated symplectic (Fig. 7; node 23).

Within Zeidae, large differences can also be seen between the last common ancestor of Zeidae (node 23) and the studied species of *Zeus* and *Zenopsis* (Fig. 7; Supplementary Figs. S3 and S4)*. Zeus faber* presents a longer maxilla with the anterior portion situated more dorsally than in the ancestral morphology. The premaxilla of *Z. faber* also displays a longer ascending process relative to the other jaw bones. This is accompanied by a relatively shorter preopercle and a smaller hyomandibula. In *Zenopsis conchifer*, the premaxilla appears relatively smaller overall. There is also a reduction in the relative size of the dentary and maxilla compared to the rest of the jaws and hyomandibular region.

Following the lineage split between Zeidae and all remaining Zeiformes, overall jaw morphology is mostly conserved through several successive ancestors (nodes 14, 15, 16, 18, 19, and 21; Figs. 6, 7). For example, node 15 (the ancestor of “cyttomimids,” Cyttidae, and Oreosomatidae), node 16 (the ancestor of “cyttomimids” and Oreosomatidae), and node 18 (the ancestor of Parazenidae, Grammicolepididae, and Zeniontidae) present very similar jaw morphologies to that of the root of the studied zeiforms at node 13 (Figs. 6 and 7).

From node 15, the lineage leading to Cyttidae diverges from the lineage to “cyttomimids” and Oreosomatidae, which share a more recent common ancestor (node 16). Cyttidae, represented here by *Cyttus australis*, presents a shorter alveolar process of the premaxilla than the ancestral morphology (Fig. 7; Supplementary Fig. S4). In addition, the anterior half of the maxilla is situated more dorsally, and the axis of the maxilla is almost parallel to the ascending process of the premaxilla.

The ancestor of “cyttomimids” and oreosomatids at node 16 shows a moderate elongation of the anterior portion of the maxilla (Fig. 7). In addition, the ascending process of the premaxilla extends toward a more anterior position than in the ancestor. In “cyttomimids,” represented here by *Capromimus abbreviatus*, the alveolar process of the premaxilla lengthens, while the dentary is shorter vertically and longer antero-posteriorly (Supplementary Fig. S4).

There are notable differences between node 16 and the last common ancestor of the studied Oreosomatidae (node 17). The ascending process of the premaxilla is more vertical in Oreosomatidae than at node 16 (Fig. 7). This is accompanied by an elongation of the anterior part of the maxilla and a relative change in orientation of the lower jaws, which become more horizontal.

Both *Oreosoma atlanticum* and *Allocyttus verrucosus* (Supplementary Fig. S4) display marked differences from their last common ancestor at node 17 (Fig. 7). In *O. atlanticum*, the preopercle is shorter along the dorso-ventral axis than at node 17, and the hyomandibula is almost perpendicular to the antero-posterior axis. *Oreosoma atlanticum* also displays a larger premaxilla with a longer ascending process than seen at node 17. In *A. verrucosus*, the maxilla is broader in its anterior section, while the premaxilla is situated more anteriorly relative to the rest of the jaws. *Allocyttus verrucosus* also displays a much broader hyomandibula.

Between node 14 and the last common ancestor of Parazenidae, Grammicolepididae, and Zeniontidae (node 18), there do not appear to be large morphological differences. Similarly, the morphological differences between node 18 and the last common ancestor of Zeniontidae and Grammicolepididae (node 21) are slight. All of these nodes are reconstructed as being similar to the ancestral condition (Fig. 7). However, larger changes occur between these nodes and the terminal species.

Zeniontidae, now containing only *Zenion* and represented in our dataset by *Z. hololepis* differs markedly in morphology from what is seen at node 21. The jaws are steeply inclined rather than terminal (Supplementary Fig. S4). The dentary of Zeniontidae is inclined steeply and is much shorter, while it remains as deep; the anguloarticular is also shortened and extends less posteriorly. The ascending process of the premaxilla makes an obtuse angle with the alveolar process and is markedly reclined posteriorly, nearly parallel to the long axis of the fishes. The long axis of the maxilla is more nearly vertical than it was in the ancestral morphology. The hyomandibula extends more posteriorly and the symplectic is situated more horizontally.

The last common ancestor of Grammicolepididae (node 22) also presents morphological differences compared with node 21. Grammicolepididae show a long dorsal extension of both the preopercle and the hyomandibula, but a much-reduced dentary and a smaller premaxilla that is situated more anterodorsally than in the ancestor. Morphological differences within Grammicolepididae are relatively small (Supplementary  Fig. S4).

Within Parazenidae, two groups diverge from node 19. The first, *Parazen pacificus*, markedly diverges from node 19 (Fig. 7) with multiple morphological changes (Supplementary  Fig. S4). For example, the dentary is elongated but also less deep dorso-ventrally, and the anguloarticular appears relatively shorter. The premaxilla has a relatively broader alveolar process, and the ascending and alveolar processes extend farther posteriorly than at node 19.

The second group consists of *Stethopristes eos* and *Cyttopsis rosea*. They share a common ancestor at node 20, which displays a morphology (Fig. 7) that is broadly similar to that of *P. pacificus*, but both *S. eos* and *C. rosea* display further morphological differences (Supplementary Fig. S4). The overall shape of the premaxilla of *S. eos* is similar to that of node 20, but the ascending process is slightly shorter, and the dorsal tip is angled more posteriorly, with the anteroventral tip of the premaxilla being more dorsal. The maxilla is shorter from its anterior tip to its posterior tip, and it is inclined ventrally. The anguloarticular extends more posteriorly, and the hyomandibula is shorter dorso-ventrally. In contrast, in *C. rosea*, the hyomandibula is taller dorso-ventrally than at node 20. Both the maxilla and the anguloarticular are longer antero-posteriorly. Also, the premaxilla is larger relative to the rest of the jaws than seen at node 20.

## Discussion

This study represents the most comprehensive examination of zeiform jaw morphologies to date. The use of µCT scanning allowed us to incorporate rare and unique specimens unavailable in previous studies. Our phylomorphospace results point to very interesting patterns of zeiform diversification.

The rapid early lineage splits leading to the zeiform crown-group families (Figs. 1 and 6) are consistent with other evidence for Early Paleogene acanthomorph radiations (e.g., Friedman 2010; Arcila and Tyler 2017; Friedman and Carnevale 2018). They are also consistent with the approximately early Eocene (50 Mya) estimated age of the zeiform crown by Ghezelayagh et al. (2021). Jaw protrusion had already evolved prior to those early lineage splits, so that all of the examined zeiform taxa share the four-bar linkage mechanism for protrusion of their upper jaws.

After the major lineages originated, their morphologies diverged, leading to a wide range of premaxillary protrusion capabilities. Differences among jaw morphologies found in the present study suggest that some zeiforms have elaborated on or emphasized that jaw-protrusion mechanism, whereas others appear to have de-emphasized it, mostly by making their jaws smaller. For example, in the morphometric results (Figs. 4 and 5), the genera *Capromimus, Cyttopsis, Oreosoma*, and *Parazen* have highly protrusible jaws as evidenced by low scores on PC1, PC2, and PC3, as well as long ascending processes of their premaxillae. These adaptations have been enhanced by evolution following the early splits in the phylogeny of zeiforms. In contrast, grammicolepidids have, relatively speaking, smaller mouths and shorter ascending processes (i.e., higher PC2 scores; Figs. 4 and 6); they thus appear to have evolved to have smaller and less protrusible jaws during the radiation of zeiforms.

*Zeus* and *Zenopsis* are an early diverging lineage from the common ancestor of almost all extant Zeiformes. Jaw protrusion had already evolved in an earlier zeiform ancestor, after which the lineages diverged, leading to a wide range of premaxillary protrusion capabilities. In the morphometric analysis, the two Zeidae have high PC1 scores (Figs. 4 and 5), but in the phylomorphospace analysis, their divergence from the ancestral condition is not as great as many other genera (Fig. 6). Similarly, the two oreosomatids *Oreosoma* and *Allocyttus* have not diverged greatly in the scores of their PC1 and PC2. However, most other lineages have diverged strongly from the near-average ancestral morphotype. These include lineages leading to *Stethopristes* and *Cyttus*, both with low values of PC1, and *Stethopristes* also with low values of PC2; these two genera have relatively smaller upper jaw bones compared with other taxa but longer and vertically taller suspensorium and posterior jaw bones. Another lineage leading to *Parazen* diverged strongly, and yet another one toward *Zenion*. These two genera display larger upper and lower jaw bones, and relatively longer suspensorium and posterior jaw bones. *Zenion* has an upturned jaw, with a horizontally long preopercle and an anterior jaw articulation (Supplementary Figs. S3 and S4). Divergent lineages also lead to *Capromimus* and even more divergently to the two grammicolepidids. Even within the single family Parazenidae there is striking divergence among the lineages leading to its three genera *Cyttopsis, Parazen*, and *Stethopristes* (Fig. 6).

In the analysis of the upper jaw, a marked divergence between *Zenion* and its sister group *Xenolepidichthys* + *Grammicolepis* is seen in morphometric PC2 and PC3 scores and in phylomorphospace PC2 scores with respect to premaxillary size and the length of the ascending process. The longer jaw, resulting from increased length of the ascending process, allows individuals of *Zenion* to slide their upper jaws farther anteriorly than those of the two grammicolepidids, resulting in greater jaw protrusibility.

Previous research conducted on zeiform morphology (e.g., Tyler et al. 2003; Grande et al. 2018) examined discrete morphological character traits and variation in overall body shape. Using morphospace results for body form from 2D landmark-based morphometrics, Grande et al. (2018) found that the early radiation of zeiform body shapes involved several closely spaced initial lineage splits, followed by major divergences among the main lineages, producing large differences in body shape among the resulting clades (Supplementary Fig. S5). The variation in body shape among zeiform subgroups (Grande et al. 2018) is paralleled in the present study by strongly divergent evolution of the jaws after initial lineage splits (Fig. 6), leading to strong differences in jaw morphology and occupation of different regions of morphospace among the major clades (Figs. 4 and 5). Interestingly, however, the major trends in jaw morphology (Fig. 6) do not closely parallel the major trends in body-form evolution (Supplementary Fig. S5).

Informative examples of differences between the earlier body-form phylomorphospace of Grande et al. (2018) (Supplementary Fig. S5), and the present jaw-based phylomorphospace (Fig. 6) are those of *Zenion* and the two genera of Zeidae: *Zeus* and *Zenopsis*. In the body-form phylomorphospace (Supplementary Fig. S5), *Zenion* was similar to *Parazen*, both being among the more fusiform zeiforms with extreme positive scores on PC1. In the present jaw phylomorphospace, *Zenion* is not closely similar to *Parazen*, but still strongly divergent, with extreme positive scores on PC1 and negative scores on PC2 (Fig. 6). In the earlier body-form phylomorphospace (Supplementary Fig. S5), the two zeid genera were convergently similar to the two grammicolepidids, *Grammicolepis* and *Xenolepidichthys.* However, in the present phylomorphospace based on jaws, the two grammicolepidids have extreme scores on PC2, whereas the two zeid genera have scores much closer to the average, ancestral condition (Fig. 6).

The present jaw phylomophospace also reflects the short initial branch lengths of the combined-evidence phylogeny of Grande et al. (2018: fig. 7; Figs. 1 and 6). A majority of zeiform taxa occupy the negative PC1 morphospace, which corresponds to a pattern of larger and more protrusible jaws. These larger, more protrusible jaws could be an important factor in the initial evolutionary success of zeiforms and could have allowed them to capture more elusive prey in their respective habitats.

The jaw phylomorphospace results also indicate evolution toward high values of PC1 (Fig. 6) between *Capromimus abbreviatus* and *Zenion hololepis*, whereas in the body-form morphospace of Grande et al. (2018), *Capromimus* was recovered as conservative, while *Zenion* was strongly divergent (Supplementary Fig. S5). This suggests that certain species have evolved to be more similar in their jaws than in their body shapes. These two genera appeared closely related in morphological phylogenies of Tyler et al. (2003), Tyler and Santini (2005), and the morphology-only analysis of Grande et al. (2018: fig. 4); therefore, the previously more inclusive family Zeniontidae (*Zenion, Capromimus*, and *Cyttomimus*) was probably held together more by its convergent morphology than by its genetic makeup.

Monophyly of Parazenidae has been debated over the years and the question still has not been fully resolved. Monophyly of Parazenidae is only supported by one discrete morphological synapomorphy and four other characters that exhibit homoplasy (Tyler et al. 2003). The synapomorphy is having one anal fin spine; most of the similar traits for this family also have to do with spine arrangement and spiny fin rays. Tyler and Santini (2005) concluded that, despite these traits, Parazenidae were not monophyletic, and that *Parazen* needed further investigation.

Grande et al. (2018) later found this group to be monophyletic in their morphology-only results, their molecular Bayesian Inference results, and in their Bayesian combined evidence phylogeny, but they were not monophyletic in molecular maximum likelihood results. Based on their combined-evidence phylogeny (Bayesian morphology + molecular), they nevertheless saw a divergence in morphospace between *Parazen*, on the one hand, and *Cyttopsis* and *Stethopristes* on the other. In the present phylomorphospace based on jaws, all three genera diverge strongly from their common ancestors (Fig. 6: nodes 19, 20). Overall, then, support for the monophyly of this clade remains qualified. Parazenidae might not be as closely related as they appear at first, but rather convergent in some morphological traits.

Oreosomatidae remain an unresolved problem largely because of poor representation in analyses, both morphological and molecular. They generally have average-sized jaws, with notable upper jaw protrusion due to the large size of their upper jaw bones. Genera within this family differ by short branch lengths early in their phylogeny, and then remain relatively conservative, as seen for *Oreosoma atlanticum* and *Allocyttus verrucosus* here (Fig. 6). Resolving their relationships and understanding trends in their jaw and body-form evolution require better representation of oreosomatid taxa and more informative data in analyses.

Overall, this study and the earlier one by Grande et al. (2018) demonstrate that morphological variation within Zeiformes evolved along at least two trajectories: body shape and jaw morphology. Evolutionary changes among genera in body form are not associated with changes among the same genera in jaw morphology, and vice versa. Possible hypotheses explaining this decoupling that merit further testing in the future include strong selection acting on the body forms, different from the strong selection acting on the jaws. The predominant mode of zeiform locomotion is known as balistiform (e.g., Dornburg et al. 2011; George and Westneat 2019), in which the fish is propelled by wavelike undulations of its dorsal and anal fin rays, while keeping its body relatively rigid, and using its paired fins for balance and pitch control. Approaching the prey stealthily in balistiform mode brings it within range, and then sudden protrusion and retraction of the jaws engulfs the prey.

Another possible explanation for the decoupling is modularity, in which jaws and bodies belong to distinct developmental and/or variational modules, allowing them to evolve along distinct trajectories (e.g., Parsons et al. 2011; Melo et al. 2016; Duclos et al. 2021; Burns et al. 2023; Evans et al. 2023; Gartner et al. 2023; Vanhaesebroucke et al. 2023). Rigorous testing for the existence of modularity or differences among patterns of modularity requires large sample sizes, which are difficult to obtain currently for Zeiformes, but might become available in the future. The apparently decoupled evolutionary trajectories of jaws and body shapes among zeiform taxa suggest that examining modularity in this group should be very enlightening.

## Supplementary Material

obae011_Supplemental_Files Includes Fig. S1, images of two representative species showing four-bar jaw-protrusion linkages, S2, a diagram showing all of the landmark positions used, S3, 2D images of scanned skulls for 12 representative zeiform species, S4, wire-frame diagrams of the jaws and suspensoria for the 12 studied zeiform species, and S5, the body-form phylomorphospace of Grande et al. (2018: fig. 7), as well as videos of three cleared-and-stained zeiform specimens illustrating jaw protrusion produced by the four-bar linkage mechanism and initiated by depression of the lower jaw.

## Data Availability

Raw data for the landmark coordinates and the R code used for morphometric and phylomorphospace analyses are archived in Dryad as DOI: 10.5061/dryad.s7h44j1d3.
