# Supplementary figures and images for: Morphological Diversity and Evolution of Jaw Morphologies in Zeiform Fishes (Teleostei, Paracanthopterygii)
Source: Integr Org Biol. 2024 Apr 15;6(1):obae011. doi: 10.1093/iob/obae011 (PMC11090498; doi:10.1093/iob/obae011)

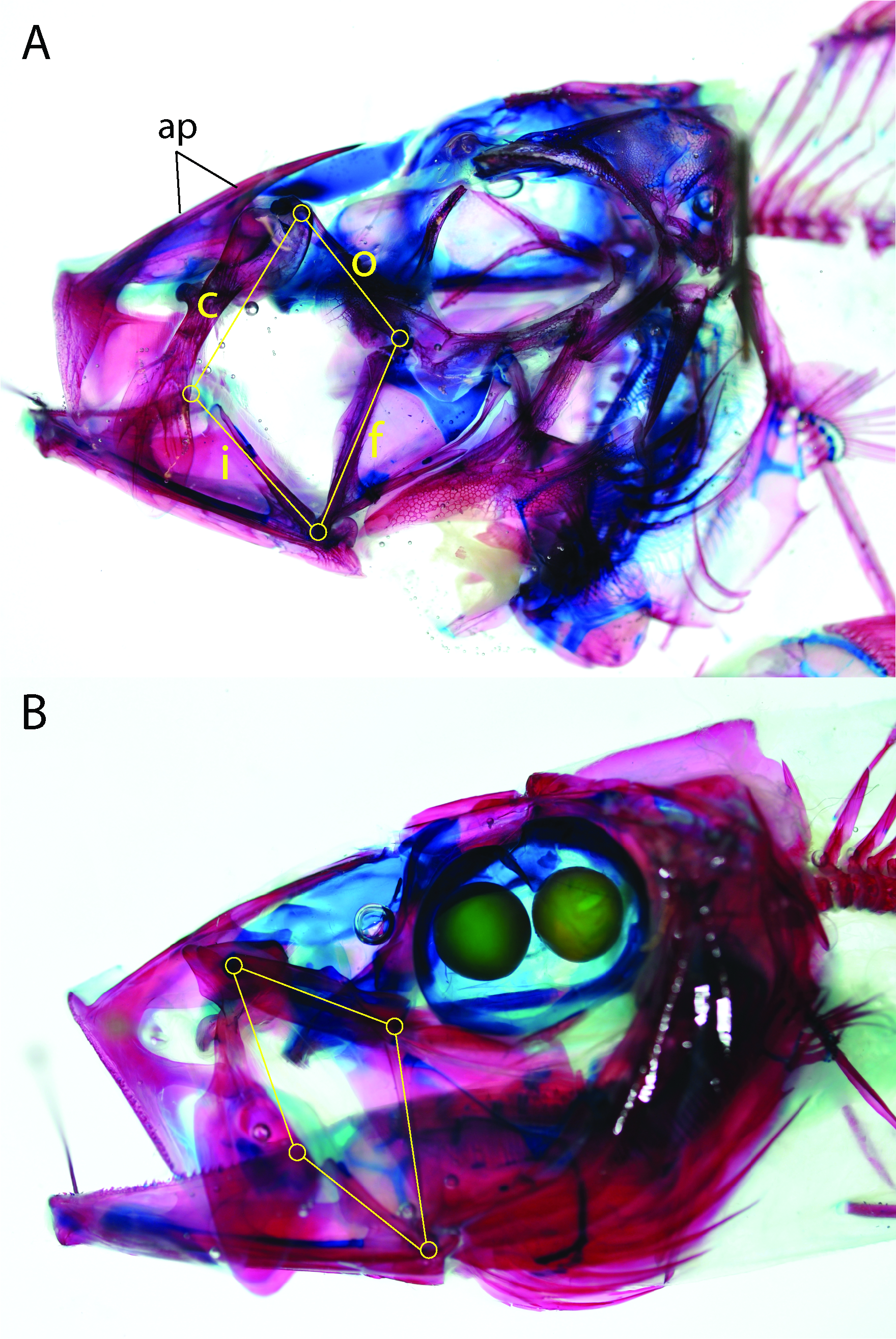

Supplement: obae011_Supplemental_Files — Includes Fig. S1, images of two representative species showing four-bar jaw-protrusion linkages, S2, a diagram showing all of the landmark positions used, S3, 2D images of scanned skulls for 12 representative zeiform species, S4, wire-frame diagrams of the jaws and suspensoria for the 12 studied zeiform species, and S5, the body-form phylomorphospace of Grande et al. (2018: fig. 7), as well as videos of three cleared-and-stained zeiform specimens illustrating jaw protrusion produced by the four-bar linkage mechanism and initiated by depression of the lower jaw. [file obae011_supplemental_files.zip › Fig. S1 Cyttopsis and Parazen four-bar linkage CMYK.tif]

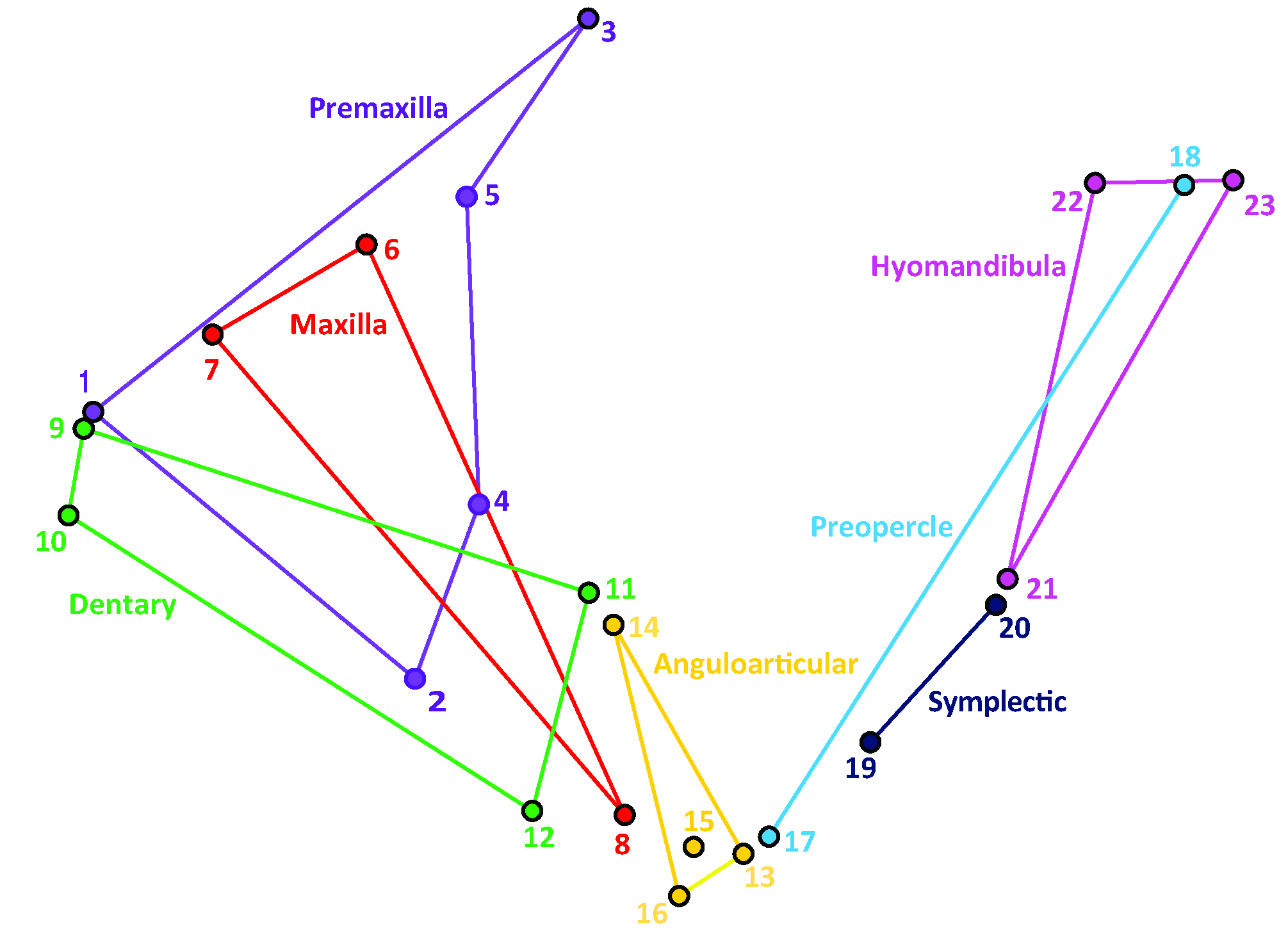

Supplement: obae011_Supplemental_Files — Includes Fig. S1, images of two representative species showing four-bar jaw-protrusion linkages, S2, a diagram showing all of the landmark positions used, S3, 2D images of scanned skulls for 12 representative zeiform species, S4, wire-frame diagrams of the jaws and suspensoria for the 12 studied zeiform species, and S5, the body-form phylomorphospace of Grande et al. (2018: fig. 7), as well as videos of three cleared-and-stained zeiform specimens illustrating jaw protrusion produced by the four-bar linkage mechanism and initiated by depression of the lower jaw. [file obae011_supplemental_files.zip › Fig. S2 wireframe diagram colored CMYK.tif]

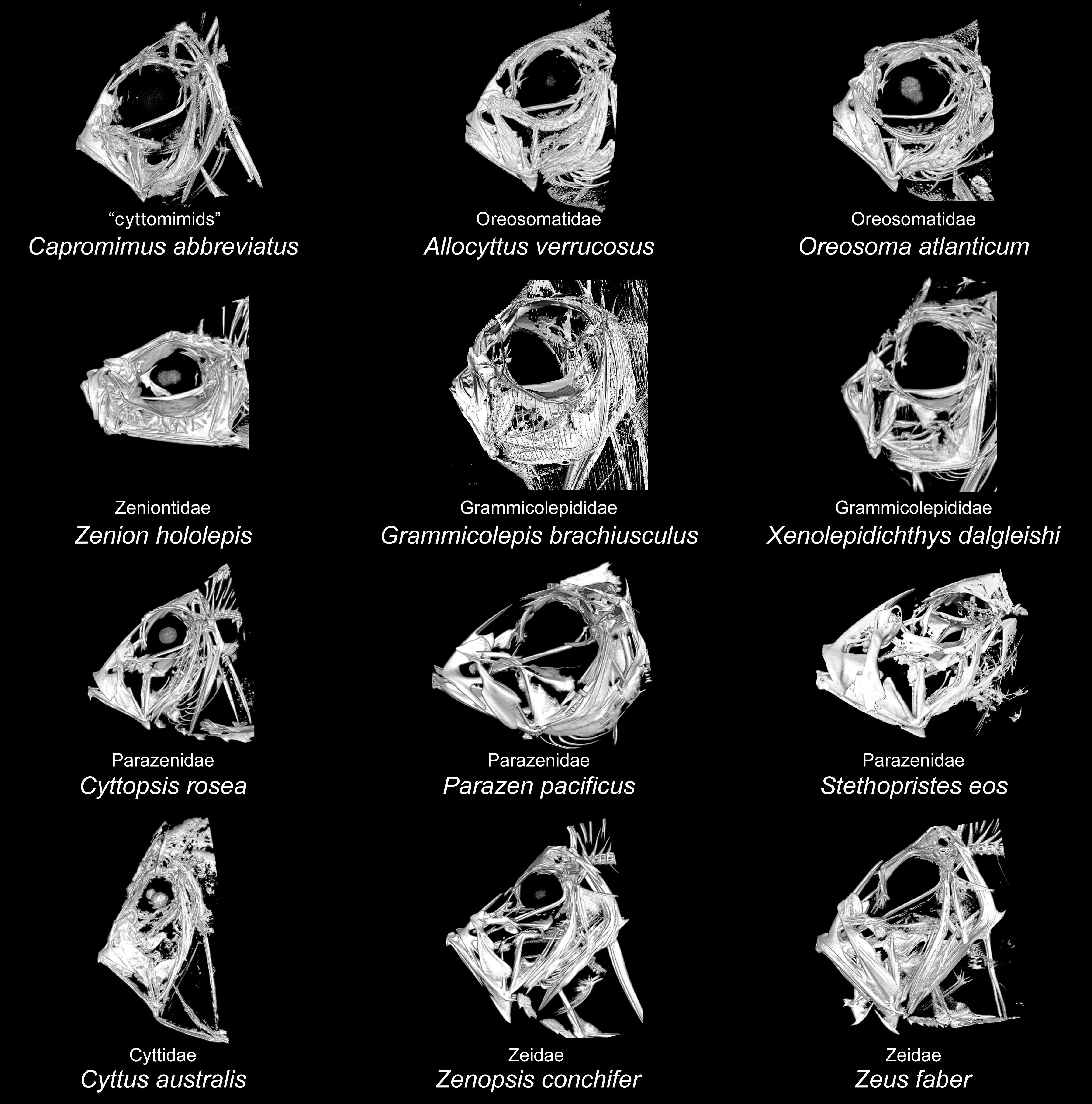

Supplement: obae011_Supplemental_Files — Includes Fig. S1, images of two representative species showing four-bar jaw-protrusion linkages, S2, a diagram showing all of the landmark positions used, S3, 2D images of scanned skulls for 12 representative zeiform species, S4, wire-frame diagrams of the jaws and suspensoria for the 12 studied zeiform species, and S5, the body-form phylomorphospace of Grande et al. (2018: fig. 7), as well as videos of three cleared-and-stained zeiform specimens illustrating jaw protrusion produced by the four-bar linkage mechanism and initiated by depression of the lower jaw. [file obae011_supplemental_files.zip › Fig. S3 2dscans of zeiform skulls greyscale.tif]

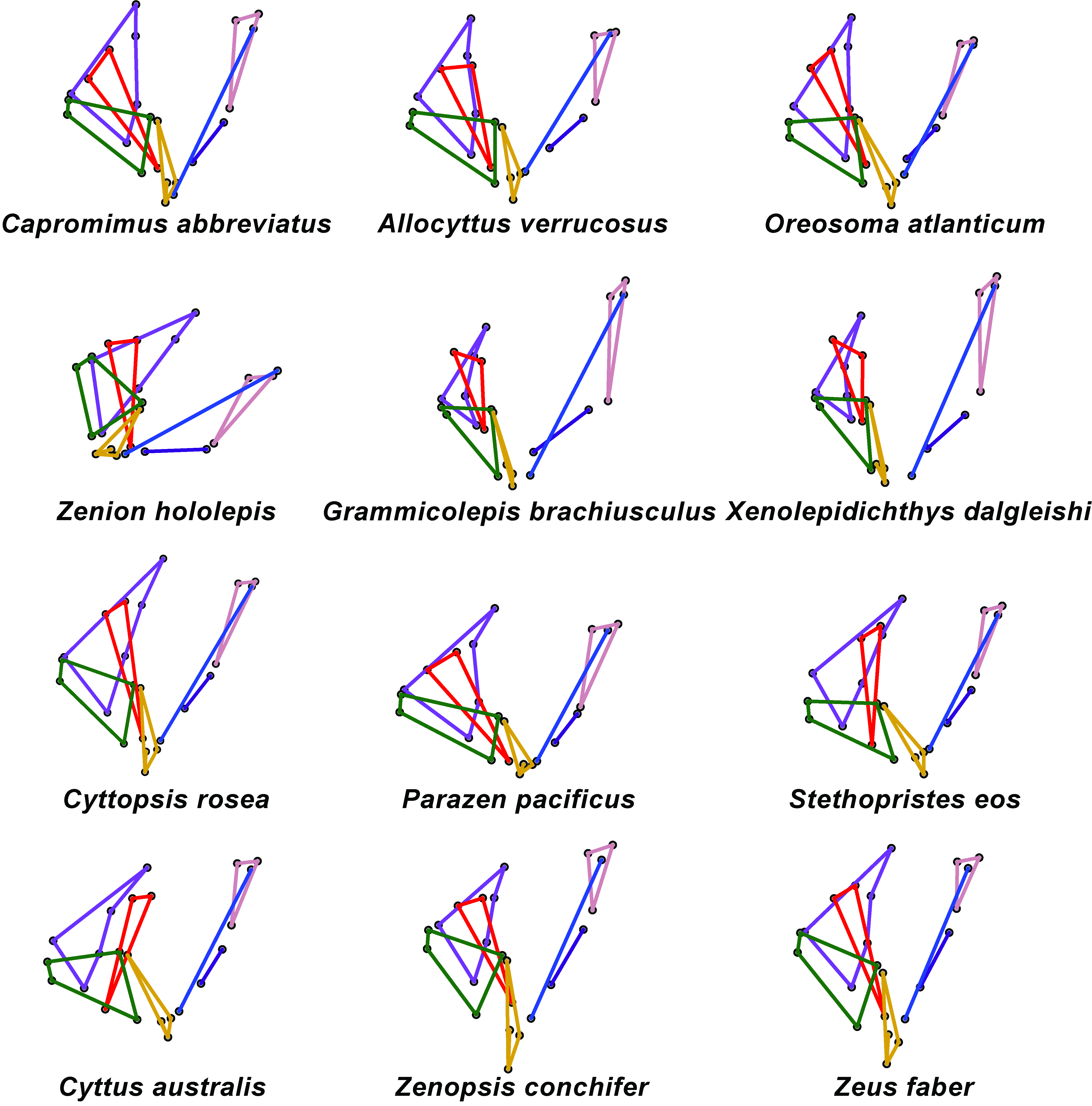

Supplement: obae011_Supplemental_Files — Includes Fig. S1, images of two representative species showing four-bar jaw-protrusion linkages, S2, a diagram showing all of the landmark positions used, S3, 2D images of scanned skulls for 12 representative zeiform species, S4, wire-frame diagrams of the jaws and suspensoria for the 12 studied zeiform species, and S5, the body-form phylomorphospace of Grande et al. (2018: fig. 7), as well as videos of three cleared-and-stained zeiform specimens illustrating jaw protrusion produced by the four-bar linkage mechanism and initiated by depression of the lower jaw. [file obae011_supplemental_files.zip › Fig. S4 wireframes for 12 studied species CMYK.tif]

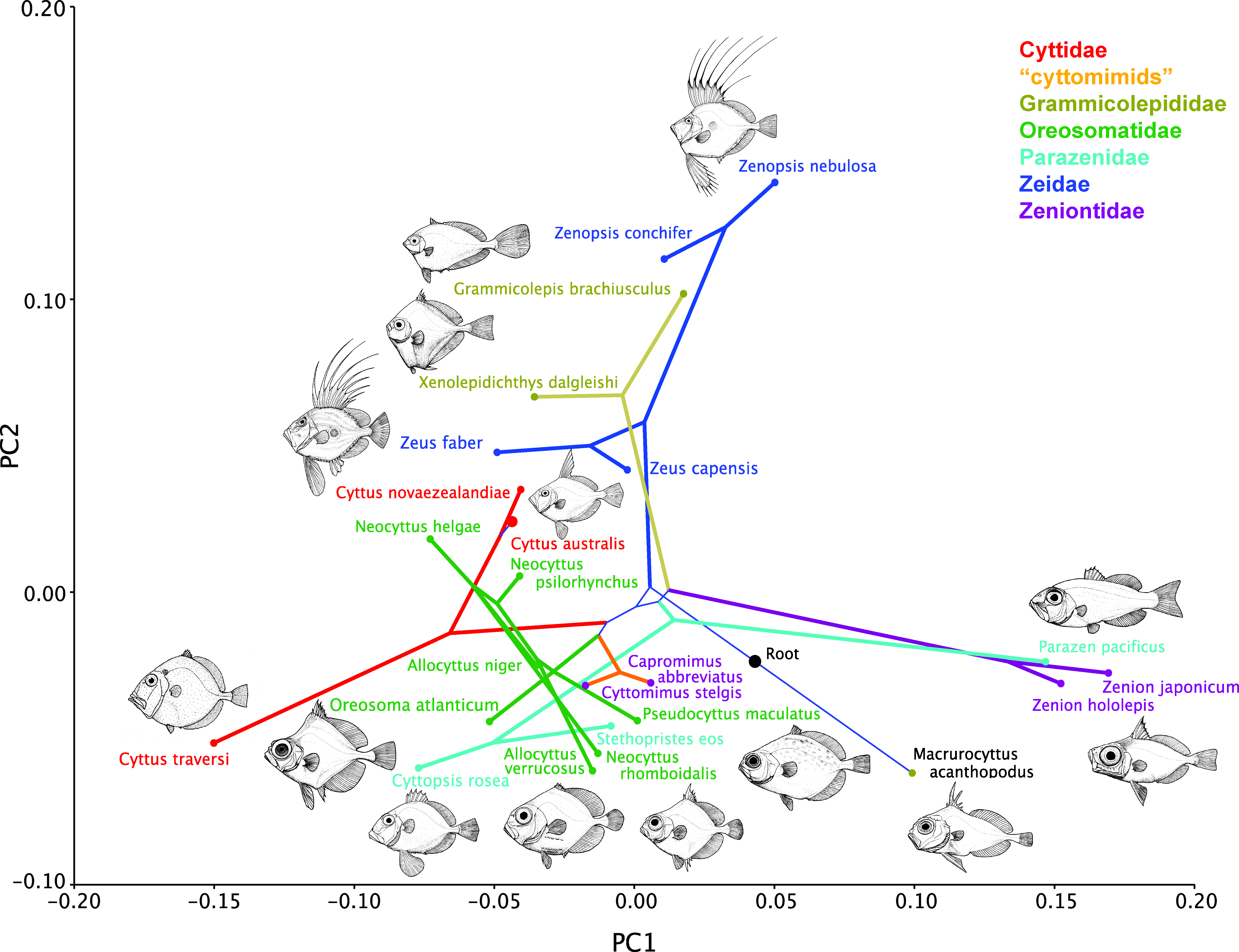

Supplement: obae011_Supplemental_Files — Includes Fig. S1, images of two representative species showing four-bar jaw-protrusion linkages, S2, a diagram showing all of the landmark positions used, S3, 2D images of scanned skulls for 12 representative zeiform species, S4, wire-frame diagrams of the jaws and suspensoria for the 12 studied zeiform species, and S5, the body-form phylomorphospace of Grande et al. (2018: fig. 7), as well as videos of three cleared-and-stained zeiform specimens illustrating jaw protrusion produced by the four-bar linkage mechanism and initiated by depression of the lower jaw. [file obae011_supplemental_files.zip › Fig. S5 Grande et al. 2018 fig. 7 body-form phylomorphospace CMYK.tif]
